# Supplementary material for: Safety and immunogenicity of VLPCOV-02, a SARS-CoV-2 self-amplifying RNA vaccine with a modified base, 5-methylcytosine
Source: iScience. 2024 Jan 22;27(2):108964. doi: 10.1016/j.isci.2024.108964 (PMC10863314; doi:10.1016/j.isci.2024.108964)
Supplement: Document S1. Figures S1 and S2 and Tables S1–S4 [file mmc1.pdf]

iScience, Volume *27*

Supplemental information

Safety and immunogenicity of VLPCOV-02, a SARS-CoV-2 self-amplifying RNA vaccine with a modified base, 5-methylcytosine

Masayuki Aboshi, Kenta Matsuda, Daisuke Kawakami, Kaoru Kono, Yoko Kazami, Takashi Sekida, Mai Komori, Amber L. Morey, Shigeru Suga, Jonathan F. Smith, Takasuke Fukuhara, Yasumasa Iwatani, Takuya Yamamoto, Nobuaki Sato, and Wataru Akahata

Figure S1. Schematic representation of the vaccine design, related to STAR methods.

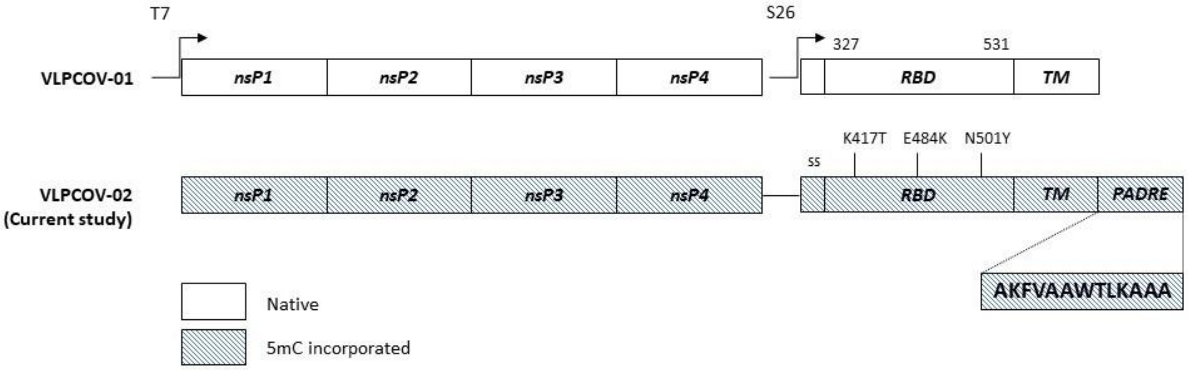

Figure S2. Study disposition flow diagram, related to Figure 1.

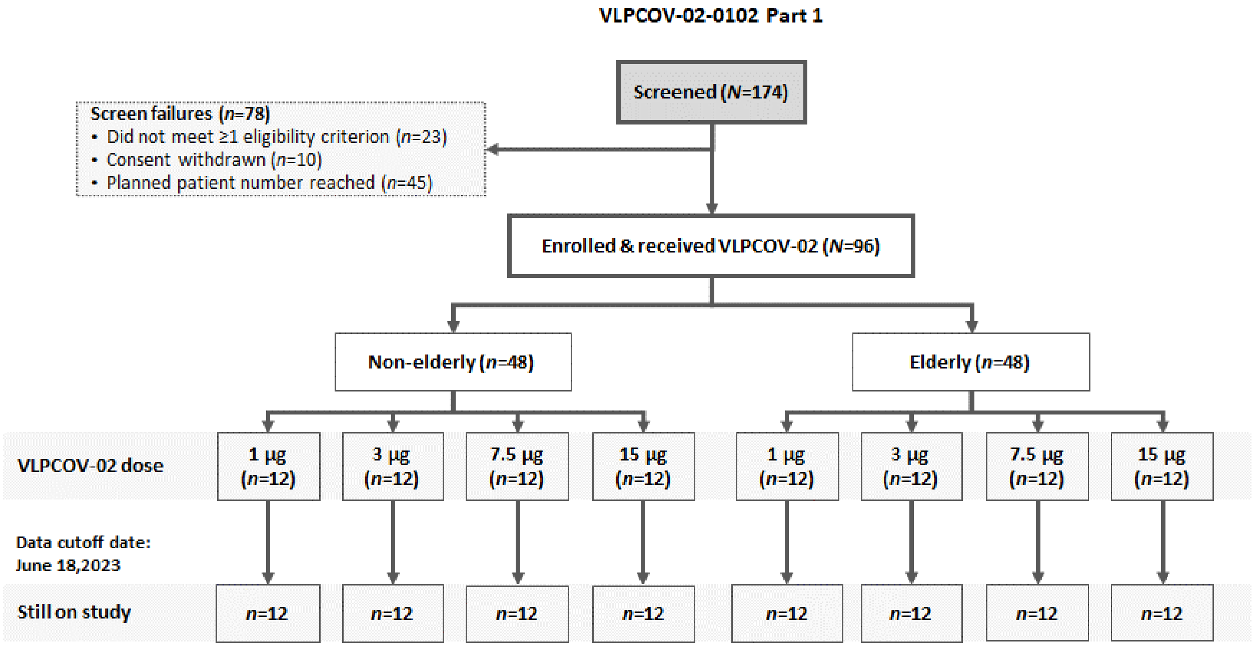

Table S1. Serum SARS-CoV-2 IgG titers[a] at baseline (day 0) and 4 weeks after vaccination (day 28) by participant, and geometric mean titer for participants in the non-elderly and elderly cohorts who received VLPCOV-02 1 µg, related to Figure 3.

**VLPCOV-02 dose**	**VLPCOV-02 1 µg**			
**Age cohort**	**Non-elderly**		**Elderly**	
**Day post-immunization**	**0**	**28**	**0**	**28**
Participant 1	4660	8580	11,900	56,100
Participant 2	1730	2700	732	6740
Participant 3	2790	22,100	6050	17,400
Participant 4	14,500	34,700	5300	37,100
Participant 5	2790	16,300	26,600	38,100
Participant 6	7960	20,000	24,400	≥80,000
Participant 7	2180	14,200	1930	4250
Participant 8	51,000	68,100	1050	3250
Participant 9	4610	30,000	56,500	51,700
Participant 0	3710	6870	≥80,000	≥80,000
Participant 11	5940	≥80,000	45,500	43,600
Participant 12	5490	24,700	14,900	61,200
**Geometric mean titer[a]**	**5,260.6**	**19,047.6**	**10,099.2**	**26,376.7**
**95% CI**	**2,928.6–9,449.3**	**10,410.7–34,849.9**	**3743.6–27,245.1**	**12,734.2–54,634.8**

[a]The maximum titer measurable was 80,000; any values ≥80,000 were treated as 80,000 for the purposes of calculating the geometric mean titers and 95% confidence intervals (CIs)

Table S2. Serum SARS-CoV-2 IgG titers[a] at baseline (day 0) and 4 weeks after vaccination (day 28) by participant, and geometric mean titer for participants in the non-elderly and elderly cohorts who received VLPCOV-02 3 μg, related to Figure 3.

**VLPCOV-02 dose**	**VLPCOV-02 3 μg**			
**Age cohort**	**Non-elderly**		**Elderly**	
**Day post-immunization**	**0**	**28**	**0**	**28**
Participant 1	51,600	68,000	≥80,000	≥80,000
Participant 2	835	13,600	2290	33,700
Participant 3	16,500	18,900	13,000	61,800
Participant 4	21,900	42,800	5620	33,300
Participant 5	33,600	52,200	9530	25,000
Participant 6	11,100	27,600	≥80,000	≥80,000
Participant 7	62,900	≥80,000	78,500	≥80,000
Participant 8	2800	9270	2090	42,400
Participant 9	44,800	53,200	1330	6770
Participant 0	1780	10,000	≥80,000	≥80,000
Participant 11	5690	18,700	4560	5200
Participant 12	53,400	63,200	34,100	≥80,000
**Geometric mean titer[a]**	**13,066.9**	**29,860.6**	**13,188.8**	**37,789.2**
**95% CI**	**5142.3–33,204.0**	**18,251.4–48,853.8**	**4828.1–36,027.4**	**20,521.1–69,588.0**

[a]The maximum titer measurable was 80,000; any values ≥80,000 were treated as 80,000 for the purposes of calculating the geometric mean titers and 95% confidence intervals (CIs)

Table S3. Serum SARS-CoV-2 IgG titers[a] at baseline (day 0) and 4 weeks after vaccination (day 28) by participant, and geometric mean titer for participants in the non-elderly and elderly cohorts who received VLPCOV-02 7.5 µg, related to Figure 3.

**VLPCOV-02 dose**	**VLPCOV-02 7.5 µg**			
**Age cohort**	**Non-elderly**		**Elderly**	
**Day post-immunization**	**0**	**28**	**0**	**28**
Participant 1	38,600	77,300	14,500	33,900
Participant 2	5260	49,300	58,800	≥80,000
Participant 3	41,700	≥80,000	3140	23,700
Participant 4	13,700	67,800	1860	16,100
Participant 5	14,900	≥80,000	25,300	47,400
Participant 6	42,800	≥80,000	8400	28,800
Participant 7	32,200	≥80,000	33,500	76,600
Participant 8	28,500	≥80,000	28,300	70,500
Participant 9	2500	50,700	11,800	21,800
Participant 0	34,700	≥80,000	2990	5230
Participant 11	3540	29,600	35,700	≥80,000
Participant 12	1260	≥80,000	14,900	24,500
**Geometric mean titer[a]**	**13,130.6**	**66,965.1**	**12,771.3**	**33,060.2**
**95% CI**	**5951.5–28,969.3**	**54,877.1–81,715.9**	**6329.3–25,770.1**	**19,714.5–55,440.4**

[a]The maximum titer measurable was 80,000; any values ≥80,000 were treated as 80,000 for the purposes of calculating the geometric mean titers and 95% confidence intervals (CIs)

Table S4. Serum SARS-CoV-2 IgG titers[a] at baseline (day 0) and 4 weeks after vaccination (day 28) by participant, and geometric mean titer for participants in the non-elderly and elderly cohorts who received VLPCOV-02 15 µg, related to Figure 3.

**VLPCOV-02 dose**	**VLPCOV-02 15 µg**			
**Age cohort**	**Non-elderly**		**Elderly**	
**Day post-immunization**	**0**	**28**	**0**	**28**
Participant 1	4060	11,700	11,200	59,700
Participant 2	5860	55,500	68,600	≥80,000
Participant 3	4180	44,000	15,500	53,600
Participant 4	27,800	76,800	74,600	≥80,000
Participant 5	26,400	62,900	30,400	36,700
Participant 6	23,900	46,800	15,800	36,800
Participant 7	66,800	≥80,000	36,300	53,000
Participant 8	4790	≥80,000	11,200	69,200
Participant 9	21,800	47,100	17,600	23,200
Participant 0	6300	31,400	≥80,000	≥80,000
Participant 11	17,700	≥80,000	2780	27,300
Participant 12	12,500	40,800	17,700	55,700
**Geometric mean titer[a]**	**12,660.6**	**49,166.9**	**21,877.2**	**50,668.1**
**95% CI**	**7062.6–22,695.8**	**34,718.8–69,627.4**	**11,820.6–40,489.4**	**38,740.9–66,267.3**

[a]The maximum titer measurable was 80,000; any values ≥80,000 were treated as 80,000 for the purposes of calculating the geometric mean titers and 95% confidence intervals (CIs).
